# Health education interventions on knowledge and self-care practices for hypertensive disorders during pregnancy: systematic review and meta-analysis protocol*

**DOI:** 10.15649/cuidarte.2495

**Published:** 2023-05-28

**Authors:** Iliana Milena Ulloa-Sabogal, Giovanny Andrés Pérez-Jaimes, Edier Mauricio Arias-Rojas, Wilson Cañon-Montañez

**Affiliations:** 1 . RN, MSN. Faculty of Nursing, Universidad de Antioquia, Medellín, Colombia. E-mail: iliana.ulloa@udea.edu.co Universidad de Antioquia Faculty of Nursing Universidad de Antioquia Medellín Colombia iliana.ulloa@udea.edu.co; 2 . RN. Nursing School, Universidad Industrial de Santander, Bucaramanga, Colombia. E-mail: giofia852@hotmail.com Universidad Industrial de Santander Nursing School Universidad Industrial de Santander Bucaramanga Colombia giofia852@hotmail.com; 3 . RN, MSN, PhD. Faculty of Nursing, Universidad de Antioquia, Medellín, Colombia. E-mail: emauricio.arias@udea.edu.co Universidad de Antioquia Faculty of Nursing Universidad de Antioquia Medellín Colombia emauricio.arias@udea.edu.co; 4 .RN, MSc. PhD. Faculty of Nursing, Universidad de Antioquia, Medellín, Colombia. E-mail: wilson.canon@udea.edu.co Universidad de Antioquia Faculty of Nursing Universidad de Antioquia Medellín Colombia wilson.canon@udea.edu.co

**Keywords:** Health education, Knowledge, Self-care, Hypertension, Pregnancy-Induced, Systematic Review, Meta-Analysis, Educación en salud, Conocimiento, Autocuidado, Hipertensión Inducida en el Embarazo, Revisión Sistemática, Metaanálisis, Educacáo em saúde, Conhecimento, Autocuidado, Hipertensáo Induzida pela Gravidez, Revisáo Sistemática, Metanálise

## Abstract

**Introduction:**

Hypertensive disorders during pregnancy are a global health problem. Health education is a strategy that provides pregnant women with knowledge and skills for self-care.

**Objective:**

evaluate the effect of health education interventions on pregnant women's knowledge and self-care practices for hypertensive disorders in pregnancy, compared to standard prenatal care.

**Materials and Methods:**

Systematic review and meta-analysis protocol. The study record can be consulted in PROSPERO (CRD42021252401). The search will be conducted in the following databases, PubMed/MEDLINE, CENTRAL, LILACS, CINAHL, EMBASE, and WoS. Additionally, clinical trial records in ClinicalTrials and grey literature in OpenGrey and Google Scholar. The search will include studies of health education intervention in knowledge and self-care practices about hypertensive disorders in pregnancy. All statistical analysis will be carried out with the Review Manager software. Data will be combined using random-effects models, binary data with odds ratios or relative risks, and continuous data using mean differences. Heterogeneity between studies will be assessed using the Q-Cochran test to measure the significance and the l2 statistic to measure magnitude.

**Discussion:**

This study will contribute to the knowledge of health interventions that are effective in guiding and educating pregnant women about the disease and self-care practices.

**Conclusion:**

The results of this study will be used to provide recommendations in the management of maternal perinatal care, that promote comprehensive care in accordance with the Primary Health Care policy.

## Resumen

**Introducción:**

Los trastornos hipertensivos durante el embarazo constituyen un problema de salud a nivel mundial. La educación para la salud es una estrategia que brinda a la mujer embarazada conocimientos y habilidades para el autocuidado.

**Objetivo:**

evaluar el efecto de intervenciones en educación para la salud sobre el conocimiento y las prácticas de autocuidado de mujeres embarazadas ante los trastornos hipertensivos en el embarazo, en comparación con la atención estándar del control prenatal.

**Materiales y Métodos:**

Protocolo de revisión sistemática y metaanálisis. El registro del estudio puede ser consultado en PROSPERO (CRD42021252401). La búsqueda se realizará en las siguientes bases de datos, PubMed/ MEDLINE, CENTRAL, LILACS, CINAHL, EMBASE y WoS. Adicionalmente, registros de ensayos clínicos en ClinicalTrials y literatura gris en OpenGrey y Google Scholar. La búsqueda incluirá estudios de intervenciones en educación para la salud sobre conocimientos y prácticas de autocuidado ante los trastornos hipertensivos del embarazo. Los análisis estadísticos se llevarán a cabo con el software Review Manager. Los datos se combinarán mediante modelos de efectos aleatorios, los datos binarios con odds ratios o riesgos relativos y los datos continuos mediante diferencia de medias. La heterogeneidad entre los estudios se evaluará mediante la prueba Q-Cochran para medir la significancia y el estadístico l2 para medir la magnitud.

**Discusión:**

Este estudio aportará en el conocimiento de las intervenciones en salud que son efectivas para orientar y educar a las mujeres embarazadas sobre la enfermedad y prácticas de autocuidado.

**Conclusión:**

Los resultados de este estudio servirán para proporcionar recomendaciones en la gestión del cuidado materno perinatal, que promuevan atención integral acorde con la política de Atención Primaria en Salud.

## Resumo

**Introdução:**

Os distúrbios hipertensivos durante a gravidez sao um problema de saúde globalA educagáo em saúde é uma estratégia que proporciona as gestantes conhecimentos e habilidades para o autocuidado.

**Objetivo:**

avaliar o efeito das intervencoes de educagáo em saúde no conhecimento e nas práticas de autocuidado das gestantes para os distúrbios hipertensivos na gravidez, em comparado com o pré-natal padráo.

**Materiais e Métodos:**

Protocolo de revisáo sistemática e meta-análise.O registro do estudo pode ser consultado no PROSPERO (CRD42021252401). A busca será realizada nas seguintes bases de dados, PubMed/MEDLINE, CENTRAL, LILACS, CINAHL, EMBASE e WoS. Além disso, registros de ensaios clínicos no ClinicalTrials e literatura cinzenta no OpenGrey e Google Scholar. A pesquisa incluirá estudos de intervencáo de educacáo em saúde no conhecimento e nas práticas de autocuidado sobre as doencas hipertensivas na gravidez. Todas as análises estatísticas seráo realizadas com o software Review Manager. Os dados seráo combinados usando modelos de efeitos aleatórios, dados binários com razoes de chances ou riscos relativos e dados contínuos usando diferencas médias. A heterogeneidade entre os estudos será avaliada utilizando o teste Q-Cochran para medir a significancia e a estatística l2 para medir a magnitude.

**Discussáo:**

Este estudo contribuirá para o conhecimento de intervencoes em saúde eficazes na orientacáo e educacáo das gestantes sobre a doenca e as práticas de autocuidado.

**Conclusáo:**

Os resultados deste estudo seráo utilizados para fornecer recomendacoes na gestáo da atencáo perinatal materna, que promovam a integralidade da atencáo de acordo com a política de Atencáo Primária a Saúde.

## Introduction

Hypertensive disorders in pregnancy (HDP) constitute a set of clinical conditions whose common factor is the presence of blood pressure figures equal to or greater than 140/90 mmHg after the 20th week of pregnancy, childbirth, or postpartum. This group of disorders is classified into four categories: chronic hypertension, gestational hypertension, preeclampsia chronic hypertension with super aggregated preeclampsia^1^. In addition, preeclampsia can lead to complications such as eclampsia and HELLP syndrome (hemolysis, elevated liver enzymes, and low platelet count)^2^.

HDP contributes significantly to maternal, fetal, and neonatal morbidity and mortality^3^. It is estimated that hypertension complicates 3% to 10% of all pregnancies, is responsible for 18% of maternal mortality^4^, 20% of fetal mortality, and accounts for 25% of hospitalizations of complicated pregnancies^5^. Therefore, HDP is public health problem globally^3^^,^^6^. The World Health Organization (WHO) states that HDP is the third cause of maternal mortality worldwide. In Latin America and the Caribbean, they are the leading cause of maternal mortality^7^. Preeclampsia is one of the hypertensive disorders with the greatest impact on maternal and neonatal health^7^. However, the prevalence of preeclampsia in Europe is 2.8%-5.2%, in Asia 0.2%-6.7%, in Africa 0.5%-2.3%, in Oceania 2.8%-9.2%, in South America and the Caribbean 1.8%-7.7% and in North America 2,6%-4,0%^3^.

HDP is associated with a high risk of physiological complications in maternal and perinatal health^4^^,^^8^. In addition, they are the major cause of prolonged maternal and neonatal hospitalizations^7^, interfering with joint mother and child, maternal bond, and initiation of breastfeeding^9^. HDP can also trigger psychological and emotional problems in mothers^10^^-^^13^, compromising parenting capacity and mother relationship with the baby^13^. In addition, they represent a significant economic burden for the family^5^, health institutions, the country, and society^14^^,^^15^.

According to Mendoza et al.^1^, the impact of HDP on perinatal maternal health has been the subject of an investigation by numerous studies aiming to determine its etiology. However, the knowledge obtained regarding the exact cause of these hypertensive conditions is limited^1^^,^^16^. Therefore, it is proposed that immunological, genetic, clinical, family, personal, obstetric, lifestyle, nutritional habits, as well as sociodemographic^16^ and psychosocial variables, contribute to the development of HDP^9^.

Studies also documented that the low quality of health care generates a relatively high burden in the incidence and complications associated with its pathology. Some of the low quality of health care practices are inadequate infrastructure, supplies, and hospital equipment^17^, the poor training, and updating of health personnel are ineffective in managing HDP^17^^,^^18^. Moreover, poor communication and explanation of pregnancy results during the prenatal consultation complicate HDP^17^.

Recent studies suggest that the high rate of maternal morbidity and mortality is associated with HDP owing to limited or non-existent knowledge, a negative attitude towards the disease, and a lack of preventive and self-care practices by pregnant women^19^. Because of this, the culture of perinatal maternal care rescues the promotion and maintenance of health, and the prevention of the disease as fundamental pillars to prevent complications derived from pregnancy^20^. A key component in health promotion and disease prevention are health education interventions, which are defined as "a set of actions aimed at informing and motivating the population to adopt and maintain healthy lifestyle habits, prevent diseases, and improve the quality of life."^21^ Thus, health education is vital in increasing the knowledge and preventive behavior of pregnant women in the face of pregnancy complications^22^. In this regard, Parsa et al.^23^ and Alnuaimi et al.^24^ stated that educational interventions are aimed at increasing women's level of knowledge regarding HDP to significantly improved perinatal maternal health outcomes. Typically, knowledge facilitates the development of care practices, early identification of signs and symptoms, and the timely seeking of medical attention.

From this perspective, the prevention, management or control of the risk of HDP could be improved through educational interventions aimed at improving women's knowledge and self-care practices during the prenatal stage. However, it has been described that intervention strategies in health education aimed at improving knowledge and preventive behaviors in the face of pregnancy problems are scarce. In addition, existing ones have not shown a reduction of risk and complications in pregnancy. Among the reasons for this, it has been identified that the means of delivery, the times and the contents of educational programs are not adequate or adapted to the conditions of pregnant women, which can affect the results of the interventions^22^.

In this sense, it has been described that health professionals, and especially nurses are an important resource in the implementation of educational programs aimed to improving the knowledge and practices of care of pregnant women before the HDP^25^. The WHO recommends delegating prenatal care to the nursing staff, as they are the professionals who not only have the knowledge and disciplinary skills in maternal care^26^, but also the most constant, direct, and active participation with women and their families at different times of prenatal care^27^.

In summary, the review of the existing literature is essential to recognize the impact of educational interventions on the knowledge and self-care practices of pregnant women before the HDP. In addition, no previous systematic review or meta-analysis has examined health education intervention studies on hypertensive pregnancy complications. Therefore, this systematic review and meta-analysis will constitute a more comprehensive, rigorous, and reliable study of the scientific evidence published to date. Therefore, the purpose of this study is to evaluate the effect of health education interventions on pregnant women's knowledge and self-care practices for hypertensive disorders in pregnancy, compared to usual prenatal care.

## Material and Methods

### Study design

A systematic review and meta-analysis oriented on the guidelines of Preferred Reporting Items for Systematic Reviews and Meta-Analyses Protocols (PRISMA-P)^28^. The study record can be consulted in the International Prospective Register of Systematic Reviews (PROSPERO) under number CRD4202125240.

### Criteria established in the study

### Types of studies designs

This systematic review will include quasi-experimental studies including a control group, and randomized controlled trials (RCT). Using the PICO strategy, we developed the guiding question to facilitate the systematic literature search: Are health education interventions on knowledge and self care practices effective for hypertensive disorders during pregnancy, compared to usual prenatal care? Table 1 presents the PICO strategy.

**Table 1:** Components of the PICO strategy

Acronym/ Definition	Description
P - Population	Pregnant women diagnosed with or without hypertensive disorders
I Intervention	Health education interventions on knowledge and self-care practices for hypertensive disorders during pregnancy
C - Comparison	Usual care / standard care / control group
O - Outcome	Level of knowledge or self-care practices for hypertensive disorders during pregnancy

### Literature sources and searches

The tracking of studies will be developed independently by two researchers and conducted in the following databases: PubMed/MEDLINE, CENTRAL, LILACS, CINAHL, EMBASE, and Web of Science (WoS). No restrictions will be made based on language, date, and year of publication. All the electronic databases will be consulted from inception until June 2021.

Searches will also be conducted to identify ongoing trials and unpublished studies from ClinicalTrials. gov, OpenGrey, and Google Scholar. In addition, references from the included studies and systematic reviews will be explored to verify the inclusion of potentially eligible studies. The search strategy was developed according to the syntax of the PubMed/Medline database and adapted for other databases. As illustrated in Table 2, the equation will be performed by incorporating MeSH terms, keywords, and entry terms, adding the boolean operators "AND/OR".

**Table 2:** Equation for the PubMed/Medline database

Number	Search terms
I	((Education[MeSH Terms]) OR (Health Education[MeSH Terms]) OR (Teaching[MeSH Terms]) OR (Patient Education as Topic[MeSH Terms])
II	((Education[Title/Abstract]) OR (Educational[Title/Abstract]) OR (Training[Title/Abstract]) OR (Literacy[Title/Abstract]) OR (Teaching[Title/ Abstract])
III	I OR II
IV	((Hypertension, Pregnancy-Induced[MeSH Terms]) OR (Pre-Eclampsia[MeSH Terms]) OR (Eclampsia[MeSH Terms]) OR (HELLP Syndrome[MeSH Terms])
V	(((("Hypertension, Gestational"[Title/Abstract]) OR ("Gestational
	Hypertension"[Title/Abstract]) OR ("Pregnancy Hypertension"[Title/Abstract]) OR ("Pregnancy-induced hypertension"[Title/Abstract]) OR (Preeclampsia[Title/ Abstract]) OR ("Pre Eclampsia"[Title/Abstract]) OR (Eclampsia[Title/Abstract]) OR ("HELLP Syndrome"[Title/Abstract]) OR (Pre-Eclampsia[Title/Abstract])
VI	IV OR V
VII	((Program[Title/Abstract]) OR (intervention[Title/Abstract]) OR (Efficacy[Title/ Abstract]) OR (Effectiveness[Title/Abstract])
Number	Search terms
VIII	((((((((Self-Management[MeSH Terms]) OR (Knowledge[MeSH Terms]) OR (Health Knowledge, Attitudes, Practice[MeSH Terms]) OR (Self Care[MeSH Terms]) OR (Blood Pressure[MeSH Terms]) OR (Body Weight[MeSH Terms]) OR (Exercise[MeSH Terms]) OR (Rest[MeSH Terms]) OR (Relaxation[MeSH Terms]) OR (Diet, Healthy[MeSH Terms]) OR (Stress, Psychological[MeSH Terms]) OR (Stress, Physiological[MeSH Terms]) OR (Medication Adherence[MeSH Terms]) OR (antioxidants[MeSH Terms]) OR (Calcium[MeSH Terms]) OR (Dietary Supplements[MeSH Terms]) OR (Treatment Adherence and Compliance[MeSH Terms])
IX	((((((((((((Self-Care[Title/Abstract]) OR ("Care, Self "[Title/Abstract]) OR (Knowledge[Title/Abstract]) OR ("Self Management"[Title/Abstract]) OR ("Management, Self"[Title/Abstract]) OR (Self-Management[Title/Abstract]) OR (awareness[Title/Abstract]) OR ("Blood Pressure"[Title/Abstract]) OR ("Body Weight"[Title/Abstract]) OR (Exercise[Title/Abstract]) OR ("Physical Activity"[Title/Abstract]) OR (Rest[Title/Abstract]) OR (Relaxation[Title/ Abstract]) OR ("Healthy Eating"[Title/Abstract]) OR (stress[Title/Abstract]) OR ("urine protein"[Title/Abstract]) OR (Antioxidant[Title/Abstract]) OR (Calcium[Title/Abstract]) OR (Nutraceutical[Title/Abstract]) OR ("Dietary Supplements"[Title/Abstract]) OR ("Treatment Adherence"[Title/Abstract]) OR ("Medication Adherence"[Title/Abstract]) OR ("Diet, Healthy"[Title/Abstract]) OR ("Self-Care"[Title/Abstract])
X	VIII OR IX
XI	III AND VI AND VII AND X

### Selection of studies

**Figure 1:**
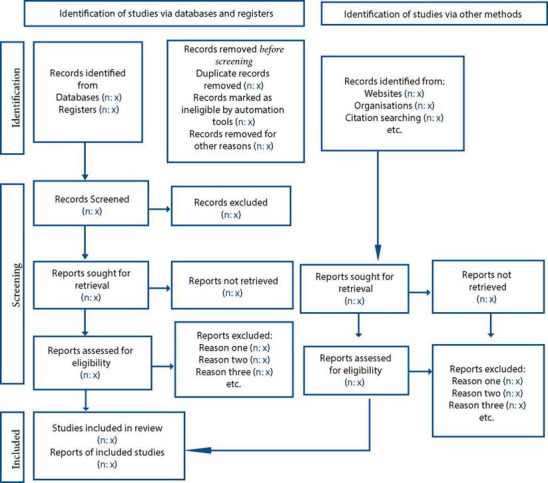
PRISMA 2020 flowchart template for systematic reviews.

According to the predefined eligibility criteria, the researchers will import the literature into the Zotero reference manager, and duplicate data will be discarded. Two researchers will independently examine the titles and abstracts to determine their eligibility, those that fail to meet the inclusion criteria will be removed at this stage. Then, full texts will then be reviewed, and the researchers decide independently whether each study meets all the inclusion criteria. Differences will be clarified by agreement or consultation with another researcher. Communication will be established with the authors to acquire more information about the study and to resolve doubts about eligibility if necessary. The number and the causes for rejection of some studies will be detailed. As illustrated in Figure 1, the selection process is shown in the Preferred Reporting Items for Systematic Reviews and Meta-Analyses (PRISMA 2020)29 flowchart.

### Classification of the quality of the studies

The studies will be evaluated using the Grading of Recommendations, Assessment, Development and Evaluation (GRADE) model^30^. The GRADE model classifies the quality of studies into the following denominations: “very low”, “low”, “moderate” and “high”. Moreover, the assessment considers the potential barriers: limitations in the design and execution, inconsistent results, imprecise results, absence of direct evidence, and publication bias^31^. Two researchers will evaluate the evidence and differences will be resolved by mutual agreement. If the disagreement continues, a third investigator will be consulted before making a final judgment.

### Appraisal of bias

The risk associated with methodological bias will be determined according to the design of the studies. Evaluations will be performed independently by the two researchers; differences will be clarified by agreement or by arbitration by the third researcher.

The risk of bias will be assessed through the Cochrane Collaboration tool (RoB 2) for RCT. The domains will be "low risk," "some concerns," and "high risk," as detailed in the tool manual^32^. The risk of bias for quasi-experimental studies will be evaluated with the ROBINS-I tool^33^. These biases will be assessed based on their level of risks: "low", "moderate", "severe", "critical risk", and "no information".

The risk of bias associated with the language of the eligible studies in this review will be addressed by the group of researchers, seeking to achieve the best understanding of the texts. Likewise, we will be accompanied by an expert librarian to help us find the best keywords and search equations in each database.

### Data synthesis

The results will be presented and synthesized through a narrative approach and thematic synthesis. To support the general description of the narrative approach, two independent researchers will record the data retrieved from the studies and list them on a box. The following information could be retrieved: standard study data, design, methodologies, description of the intervention, and outcome measures. The data will then be compared, and the researchers will discuss the differences until consensus is reached or by consultation with a third researcher. When ambiguous, contradictory, incorrect, or missing data is identified, the researchers will contact the main authors by e-mail to clarify or correct the omitted data.

### Statistical analysis

The information will be grouped and summarized in a meta-analysis with Review Manager 5.4 software. Data will be combined using random-effects models, binary data with odds ratios (OR) or relative risks (RR) and for continuous data using mean differences (MD). The data will be reported with 95% confidence intervals. The heterogeneity of the studies will be evaluated by the Q-Cochran test to measure the significance and using the l2 statistic to measure magnitude. Heterogeneity will be considered acceptable if the 12 value is less than 50% (12 < 50%) and moderate if the 12 is greater than 50% (l2 > 50%). In case of heterogeneity, the probable causes in the studies will be identified and the subgroup analysis explored, based on follow-up time and the scope of intervention.

This review will consider the primary outcome the level of knowledge of pregnant women about HDP. The secondary outcome is the self-care practices of pregnant women in the management, prevention or control of the HDP risks.

### Ethical aspects

This study will be carried out from meta-analysis methods based on information from existing primary studies. In addition, the identity of the participants will not be included in the analysis of the information. Thus, the approval of the ethics committee was not required for this study.

## Discussion

Studies have described that the lack of knowledge and self-care practices of pregnant women contribute to the increased incidence of HDP^19^^,^^34^. Moreover, the scientific evidence supports the practice of perinatal maternal care and the development of appropriate, effective, and safe health strategies aimed at educating pregnant women regarding HDP^22^. Thus, health professionals in prenatal care institutions should implement educational interventions aimed at improving the knowledge of pregnant women about the definition, classification, signs, symptoms, risk factors, complications, diagnosis, treatment, and care practices for HDP^22^^,^^34^^-^^36^. The outcomes will ultimately help women to make informed health decisions and make timely use of available health resources^36^, prerequisites for reducing maternal, fetal, neonatal morbidity, and mortality attributed to HDP^37^.

The finding of this study will provide solid data for clinical practice, policies and programs in perinatal maternal health and improve women's care during the prenatal stage. To date, no prior review has been published to establish the efficacy of educational interventions on pregnant women's self care practices and knowledge regarding HDP. Therefore, the main challenge of this study will be to synthesize the current state of the available scientific evidence on the subject, which will allow the understanding of intervention strategies in health education that significantly impact knowledge and self-care practices on HDP in pregnant women.

### Limitations

Possible limitations are a high degree of heterogeneity that studies may present, related to differences in the interventions and measurement outcomes. In addition, results and/or report measures may be inconsistent or incomplete. Thus, lead authors may be contacted to clarify or obtain missing information. Other limitation could be related to the eligible studies that are in a language of low proficiency by the research group, for which a reliable translation will be sought according to the scope and logistical capacity of the researchers.

Finally, the quality of this systematic review may be compromised by the implicit bias of the included evidence. To minimize, appropriate tools will be applied to detect each bias, and the attributes of studies will be critically explored, as described in the method section.

## Conclusion

Lack of knowledge and self-care practices about HDP can have short, medium, and long-term irreversible consequences on the well-being of pregnant women and their unborn children. Thus, health education during pregnancy is an essential component of perinatal maternal care. Health education facilitates the acquisition of knowledge of pregnant women, assisting them to develop caring skills that will enable them to reduce the risk of developing HDP and promote a healthy pregnancy.

From this perspective, the scientific evidence of this review will disseminate effective educational strategies for the self-care practices about HDP that promote comprehensive care from the Primary Health Care strategy. In addition, it will provide guidelines for future research, contributing to the design and implementation of health education interventions about HDP.
